# Ultrasonic Energy and Data Transfer through a Metal—Liquid Multi-Layer Channel Enhanced by Automatic Gain and Carrier Control

**DOI:** 10.3390/s23104697

**Published:** 2023-05-12

**Authors:** Raphael B. Pereira, Arthur M. B. Braga, Alan C. Kubrusly

**Affiliations:** 1 Center for Telecommunication Studies, Pontifical Catholic University of do Rio de Janeiro, Rio de Janeiro 22451-900, Brazil; raphael@pwm.com.br; 2 Department of Mechanical Engineering, Pontifical Catholic University of Rio de Janeiro, Rio de Janeiro 22451-900, Brazil; abraga@puc-rio.br

**Keywords:** ultrasonic, communication, frequency modulation, nonintrusive communication channel, acoustic channel, metal-fluid layers, energy harvesting

## Abstract

Ultrasonic communication and power transfer are attractive solutions when conventional electromagnetic-based or wired connections are unfeasible. Most ultrasonic communication applications concern a single-solid barrier. Nevertheless, some relevant scenarios can be composed of several fluid—solid media, through which communication and power transfer are intended. Due to its multi-layer nature, insertion loss and, consequently, the system efficiency considerably decrease. This paper presents an ultrasonic system capable of simultaneously power transferring and transmitting data through a set of two flat steel plates separated by a fluid layer using a pair of co-axially aligned piezoelectric transducers on opposite sides of the barrier. The system is based on frequency modulation and adopts a novel technique for automatic gain and automatic carrier control. The modems used herein were developed specifically for this application, rendering the system able to transfer data at a rate of 19,200 bps, using the frequency shift keying (FSK) modulation scheme and simultaneously transferring 66 mW of power through two flat steel plates (5 mm) separated by a fluid layer (100 mm), which completely supplied a pressure and temperature sensor. The proposed automatic gain control allowed a higher data transmission rate and the automatic carrier control reduced power consumption. The former reduced the transmission error from 12% to 5%, while the latter reduced the global power consumption from 2.6 W to 1.2 W. The proposed system is promising for monitoring applications such as oil wellbore structural health monitoring systems.

## 1. Introduction

The ability to transfer energy and data through metal walls in a nonintrusive way is a solution that has great potential for several monitoring systems, such as in volatile environments [1,2], high-pressure boilers [3] and aircraft fuselage [4]. Electromagnetic-based wireless transfer methods are ineffective when one or more parts (transmitter or receiver) are installed within metallic housings [5] due to the Faraday cage effect [6,7]. A possible solution is communicating through cables, which, however, requires one to penetrate the solid layers in order to insert electric cables or connectors, being therefore unfeasible in some cases such as for hulls of ships or submarines and containers and pressure vessels [8,9]. Thus, several scenarios require a solution capable of transmitting energy and data by noninvasive means.

Based on the above, the use of ultrasonic waves to transmit data and energy becomes, therefore, an attractive alternative in such scenarios. Ultrasound has been extensively employed for transmitting energy and data through metallic walls [10,11,12,13,14]. The channel usually consists of a single metallic layer with thicknesses ranging from 1.6 to 304.8 mm [9,15] with two coaxially aligned piezoelectric transducers. Different modulation methods, such as ASK [1,16,17,18], FSK [19], MFSK [20], OFDM [9,16] and QPSK [21] were used, with transmission rates ranging from 1 kbps to 17.37 Mbps.

Some practical scenarios, however, present channels that are naturally composed of multiple layers, such as oil wellbore [22,23]. In such cases, the propagation phenomenon is further complicated. Specifically regarding the amount of transmitted energy, Leckey et al. [24] showed that the damping coefficient and frequency-dependent attenuation significantly affect the waveform and amplitude of the received signals in multilayered composite plates. Other types of complex media have also been investigated. For instance, Thierry et al. [25] presented a wave propagation model in two-dimensional periodic textile composites that allows for the analysis of the interaction between the wave and textile fibers. The results showed that the model can be useful in predicting the behavior of composite structures and in optimizing their design to improve their strength and durability. Maio and Fromme [26] studied ultrasonic waves in laminated composites, under various conditions, which is useful for modeling complex geometries. In addition, the ultrasonic channel usually presents non-negligible damping. Damping can significantly attenuate the amplitude of ultrasonic waves, limiting their propagation distance and reducing the amount of energy that can be transmitted, leading to poor SNR (signal-to-noise ratio) and limited detection sensitivity [27,28]. Fewer works have addressed, however, ultrasonic communication in challenging acoustic channels, such as those composed of two or more layers of different materials [1,29]. In [29], one was able to transmit across a two-layer channel with simultaneous energy and data rates of 50 W and 4.4 Mbps across a channel composed of two steel layers with thicknesses of 15.97 and 10.92 mm separated by an 88.3 mm water column.

Here, we present a system based on frequency modulation [19] for communication through three layers, namely, metal—liquid—metal. In order to overcome the aforementioned challenging conditions, an automatic control system to dynamically adjust the receiver amplifier gain and the transmitted carrier intensity was developed in order to reduce data transmission error and the overall power consumption of the system. Both approaches were implemented at the software level, not burdening the cost of the acoustic modems. The reception amplifier gain of the automatic control was selected by using a digital technique that dismisses the need for an analog-to-digital converter, which is commonly used in this type of application [30,31]. The automatic carrier amplitude control, also proposed here, consists of controlling the carrier based on receiver feedback, improving, therefore, the overall performance of the system. The rest of the paper is organized as follows: Section 2 presents the concepts used in the research, describing how information and energy are transmitted through the acoustic channel, as well as describing the two new methods to increase the efficiency of the system; Section 3 shows the electronic circuit developed and describes the structure of the system; Section 4 presents the experimental results; and finally, Section 5 concludes the paper in addition to discussing the obtained results.

## 2. Ultrasonic Energy and Data Transmission System Based on Frequency Modulation

**Figure 1:**
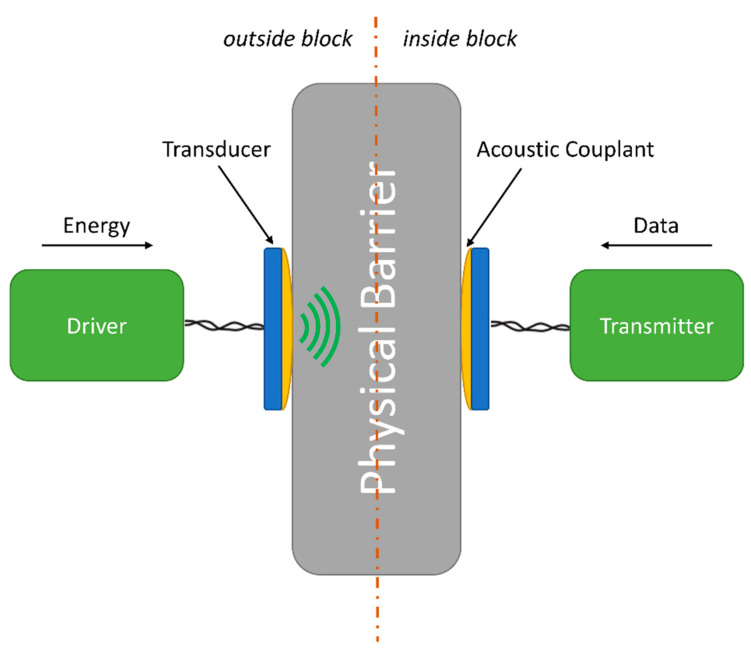
Diagram of the ultrasonic data and energy transmission system. It consists of outside and inside blocks. The outside block is responsible for sending energy to the inside block by means of ultrasonic waves. The inside block is responsible for modulating the acoustic impedance of its transducer as digital data. The driver generates the electrical signal to be applied to the transducer; the transducer converts the electrical signal into mechanical vibration and vice-versa; the transmitter modulates the transducer’s acoustic impedance by short-circuiting its terminals; the physical barrier acts as the acoustic channel.

The system addressed in this paper is composed of two main blocks, namely outside and inside blocks, adopting a concept similar to that used by Shoudy et al. [14]. The former supplies energy and receives, processes and stores data coming from the latter. The inside block can include sensors whose data are transmitted to the outside block; all energy used in this block comes exclusively from the outside block. This technique allows instruments to be installed in hard-to-reach locations, enabling monitoring of environments where the direct supply of energy or even the replacement of batteries, as a source of energy, would be unfeasible [32,33,34]. The schematic diagram of the ultrasonic transception system is shown in Figure 1.

The system operation can be summarized as follows. An electric signal produced by a driver block (left-most block of Figure 1) is applied to the transducer in the outside block. The transducer converts this electrical signal into an acoustic wave, which propagates within the acoustic channel formed by the physical barrier space. Within the latter, one is subject to acoustic-related phenomena such as damping [27,28], dispersion [35] mode-conversion [36,37] and scattering [28]. The resultant effect is manifested by a reduced amount of acoustic signal reaching the transducer in the outside block, on the right side of Figure 1, which is converted to an electrical signal and which can overall be measured by the system insertion loss [17,18]. In this way, electrical energy is transferred from the inside block to the outside block [1]. In order to simultaneously transmit data from the inside block to the outside block, one can change the electrical impedance of the inside block transducer’s terminals [2,14,38]. To that end, the transmitter block has an internal switch that, according to the modulating data, short-circuits the inside block transducer terminals, which, consequently, changes the acoustic impedance of the transducer. Therefore, the acoustic reflection coefficient also changes, which alters the amplitude of the reflected acoustic waves that are sent back to the outside block [18]. The modulating data can be detected by the outside block driver block (Figure 1) and interpreted as a digital signal [18].

Here, we implemented such an acoustic channel between the outside and inside blocks in order to transfer energy from the outside block to the inside block whilst simultaneously sending data from the inside block to the outside block. The system presents a similar structure to [1,16,17,18], but those use ASK (amplitude-shift keying) as the modulation method, whilst here a frequency modulation scheme was adopted. With this type of modulation, for each binary symbol to be transmitted, a different frequency is used to modulate the transducer in the inside block. The modulating frequencies used herein were 50 kHz (bit one) and 40 kHz (bit zero), whereas the carrier frequency was set approximately to 1 MHz. Frequency modulation offers advantages when compared to amplitude modulation, such as ASK or OOK (on—off keying); for instance, it allows higher immunity to noise and a lower probability of errors in the demodulation process [39,40,41].

In this approach, the electrical energy available at the inside block’s transducer is used to power supply to the electronic circuit within this block. This circuitry drives a sensor and generates two FSK frequencies to be applied to the transducer in order to transmit the data. This makes the inside block completely isolated and self-sufficient in terms of energy, not depending on external energy sources such as batteries, which need to be replaced from time to time. The demodulation that takes place in the outside block is performed by an electronic circuit able to measure the duration of the pulses contained within the reflected acoustic wave by the outside block transducer. By measuring the pulse duration, it is possible to calculate the signal frequency and interpret it accordingly.

### Automatic Gain Control and Automatic Carrier Control Algorithms

Aiming to enhance the system performance, either regarding data transmission or energetic efficiency, two algorithms were developed. The first automatically controls the amplitude of the carrier transmitted from the outside block to the inside block. The second controls the gain of the receiver amplifier in the outside block. Due to the inherent nonstationary nature of the acoustic channel (e.g., temperature variation), the gain necessary to detect the received signal needs to be controlled in order to allow adequate signal detection.

The automatic gain control consists of an algorithm that determines if the signal received by the outside block circuit is amplitude-saturated or, otherwise, too low. Since the sensor information data transmitted from the inside block through the acoustic channel are periodic, the control system has a counter that is reset every time the input signal at the demodulator circuit (on the outside block) switches between the high and low levels. If the input signal stays high-level for a time interval longer than the duration of one bit, the algorithm detects that there has been saturation and the outside block microcontroller reduces the amplification gain of a PGA (programmable gain amplifier), which is an electronic amplifier whose gain can be controlled externally by another signal [42]. In that case, the microcontroller in the outside block sends a message to the PGA circuit to select the desired gain. The greatest advantage of the proposed technique is that a single hardware is used to perform all the detection and demodulation processes, eliminating, therefore, a possible addition of processing modules based on an analogic electronic, which may involve component tolerance and/or sensitivity of the electronic project to noise, as a way to measure the level of the input signal and adjust the necessary gain so that the circuit could detect and demodulate the data transmitted [43,44].

**Figure 2:**
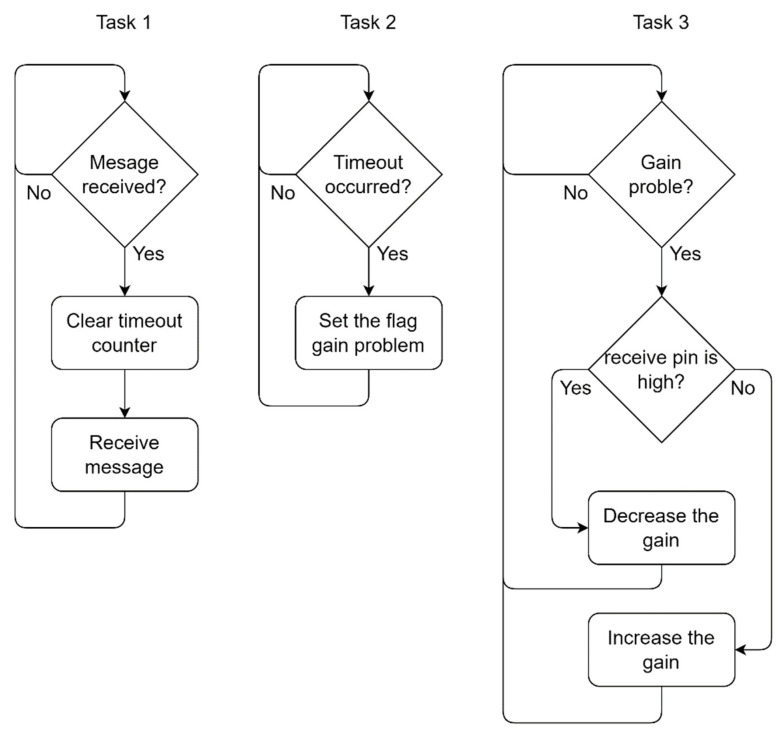
Automatic gain control algorithm tasks. Task 1 is responsible for checking if a message is received within the predefined timeout interval. Task 2 checks if a timeout occurred. Task 3 checks the Gain problem flag.

Figure 2 illustrates the flowchart of the algorithm that performs the automatic gain control. To accomplish this task, the algorithm was divided into three tasks that run concurrently. The first (Task 1) constantly checks whether any new message is about to be received and, if so, clears the flag that indicates a reception timeout. The second (Task 2) periodically checks if a timeout has occurred and, if true, it increments the gain, signaling a problem receiving data. The third (Task 3) checks the status of the gain problem flag; if it indicates that there was a problem receiving the data, the status of the receive pin is verified. This check is carried out as follows: if the receive pin of FSK modulation is at a high level (5 V), or indicates a PGA saturation, then the system decreases the PGA gain; if this pin is low (0 V), this indicates possible excessive loss channel, so the system increments the PGA gain.

**Figure 3:**
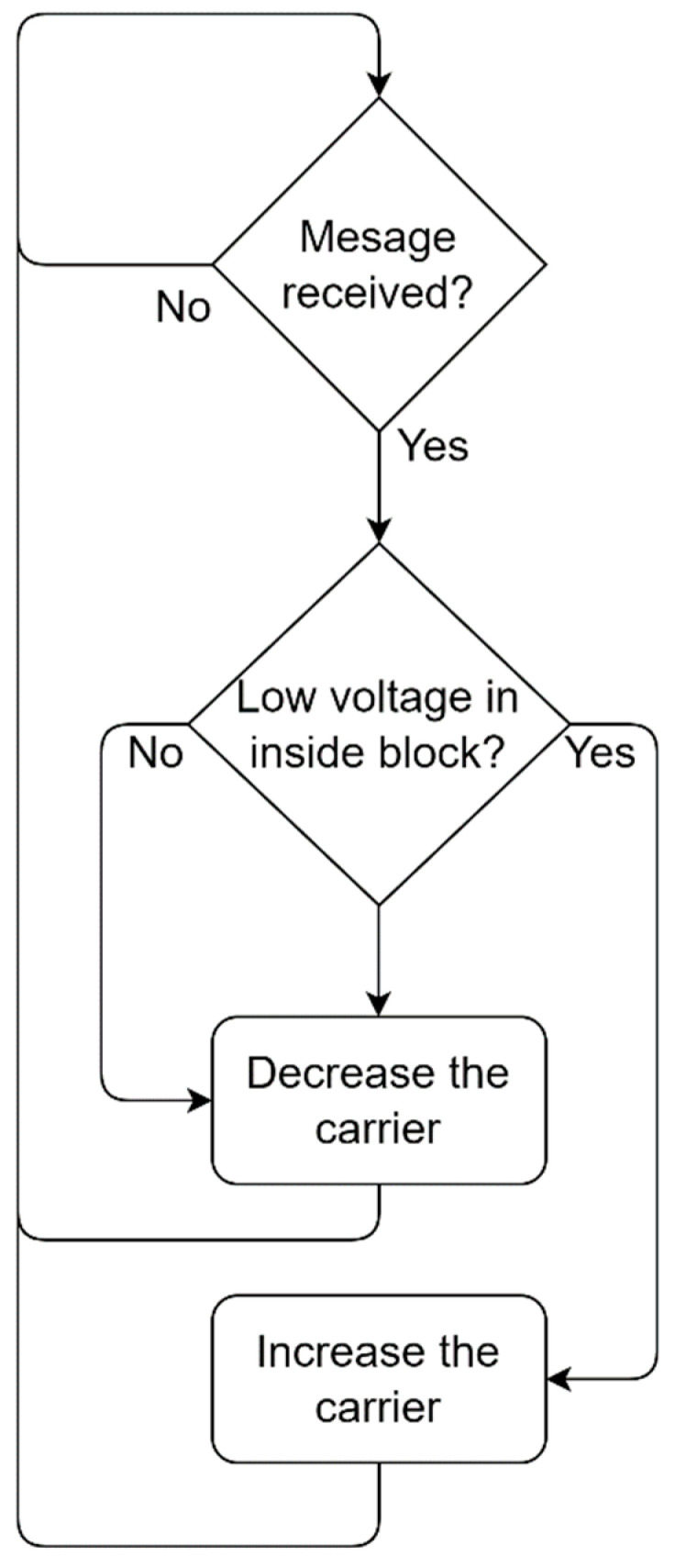
Automatic carrier control algorithm implemented in the microcontroller in order to control the carrier level transmitted from the outside to the inside block.

With the goal to reduce the total energy consumption of the system, an algorithm capable of automatically regulating the amplitude of the carrier that is transmitted from the outside block (active side of the system) to the inside block (passive side) was implemented. Unlike conventional systems that transmit a carrier with a fixed amplitude, which may transmit more energy than what is used [45,46] or systems that control the carrier but do not use it as a power source to other parts of the system [29,47], in the proposed system, an algorithm calculates the carrier level that will be applied to the outside block transducer as a function of the voltage level being delivered to the inside block circuitry. Within each transmitted symbol, a microcontroller in the inside block sends back, along with the transmitted data packet, the value of the supply voltage that was received. With this piece of information, the outside block can decide whether to alter the carrier level. If the voltage at the inside block is lower than expected, the outside block microcontroller increases the power of the carrier applied to the outside block transducer. In this way, the circuit always operates with a minimum energy level that ensures the supply voltage level of the inside block circuitry is stable and within established limits. A flowchart of the algorithm is shown in Figure 3.

## 3. Developed Circuitry

**Figure 4:**
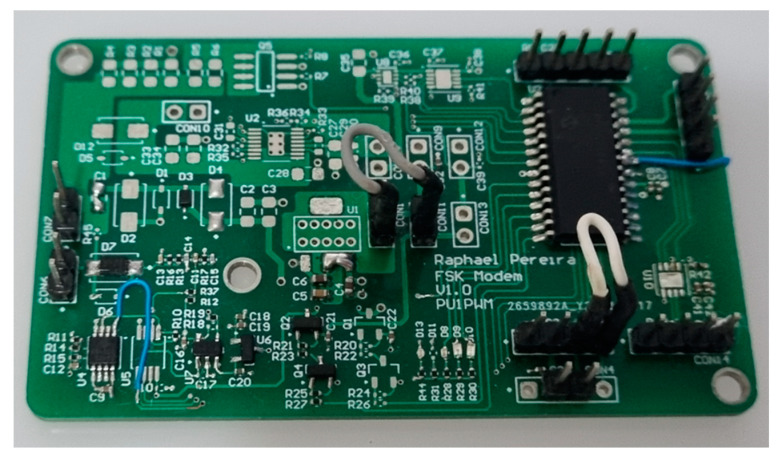
Designed modem circuitry as inside or outside blocks. This one is configured as an outside block.

In order to make the system more flexible, a single card capable of fulfilling all the functions of both a modulator and demodulator was developed. Hence, it carries within itself the possibility of receiving or transmitting signals. Thus, only one electronic board was designed, where only the components of the respective functionalities were assembled. The board is shown in Figure 4.

**Figure 5:**
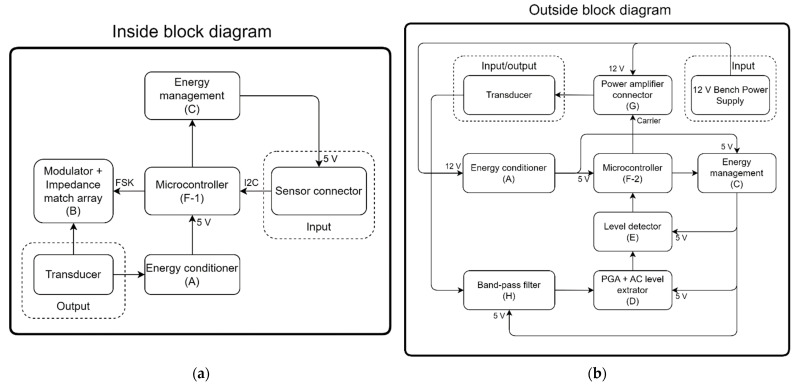
Inside (**a**) and outside (**b**) block diagrams with the main components, voltage and signals transferred within each block.

Figure 5a shows the general diagram of the inside block subcircuits. The inside block circuit operates as follows. The energy conditioner (A) is responsible for stabilizing and regulating the voltage level from the signal received at the transducer. Then, it powers the microcontroller (F-1), which activates the power level for the temperature and pressure sensor (l) through the energy manager (C). The temperature and pressure sensor (l) sends data to the microcontroller (F-1) through an I2C communication protocol. Periodically (every 500 ms), the microcontroller (F-1) sends the sensors’ data and the level of its supply voltage to the modulator (B), which applies the frequency-modulated signal to the transducer of the inside block.

Figure 5b shows the general diagram of the outside block subcircuits, which starts to work when the microcontroller (F-2) is powered by the power conditioner (A). From this moment on, the microcontroller (F-2) starts the process of generating the carrier, which is sent to the power amplifier (G). The carrier is then applied to the outside block transducer. Simultaneously, the microcontroller (F-2) acts, whenever necessary, on the programmable gain amplifier (PGA) + AC level extractor (D) subcircuits, where the AC level extractor removes the DC offset and the carrier portion from the data signal, while PGA amplifies the signal before being applied to a voltage comparator (Level detector (E)), which converts this signal into a digital signal that can then be decoded. The latter controls the gain of the PGA. The signal sent by the inside block is received by the outside block transducer and applied to a bandpass filter (H), which extracts the carrier present in the signal, producing the baseband signal. The baseband signal is sent to the PGA + AC level extractor (D), which amplifies and separates the AC part of the signal. This signal, composed only of the AC component, is applied to the level detector (E), which transforms it into a square wave that is then fed into the microcontroller (F-2) in order to be demodulated. The demodulation process performed by the microcontroller (F-2) on the outside block (Figure 5b) is obtained by measuring the frequency of the signal source by the Level detector (E). The detected frequency determines if the signal represents a high- or a low-level bit of a message previously transmitted from the inside block.

## 4. Experimental Validation

**Figure 6:**
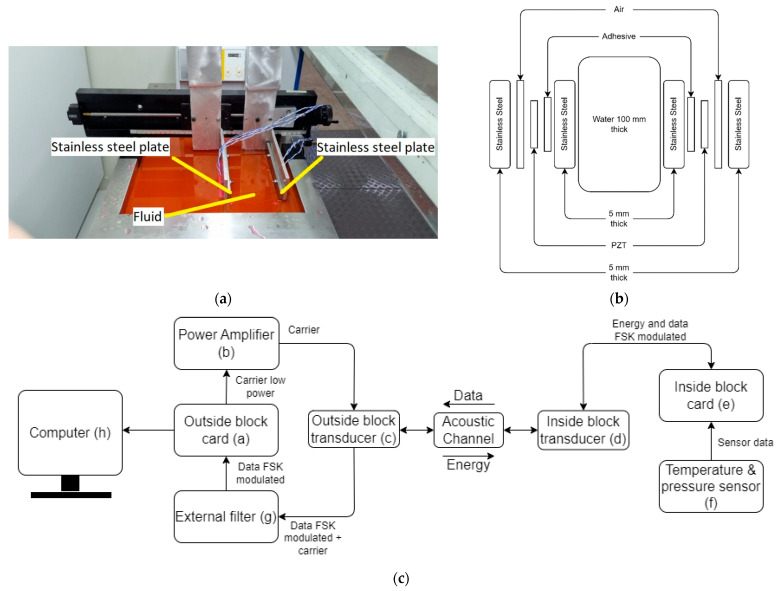
Experimental setup consisting of two transducers axially aligned, mounted in two 5 mm-thick steel walls separated by a 100 mm fluid column. Photograph of the experiment in the water recipient (**a**), corresponding schematic diagram of the multilayer acoustic channel (**b**) and detailed diagram of the experimental setup with components and connections (**c**).

The system was experimentally assessed with the setup shown in Figure 6. The acoustic channel is composed of two 5 mm-thick stainless steel blocks with fluid in between. Both stainless steel blocks were submerged in a water recipient (LAUDA^®^ Model Master EDITION X (LAUDA, Lauda-Königshofen, Germany)) with temperature control. The recipient was filled with PARAFLU^®^ and distilled water mixture in a proportion of one part of coolant fluid to five parts of distilled water in order to prevent oxidation of the metallic walls, which was kept at 25 °C. Two piezoelectric ceramic transducers with dimensions of 70 mm × 25 mm × 2 mm were bonded using an Araldite epoxy adhesive from Hunstaman^®^ (Hunstaman, The Woodlands, TX, USA) to the external surface of the steel blocks. A photograph of the setup is shown in Figure 6a and a schematic diagram illustrating the composition of each part of the acoustic channel is shown in Figure 6b. A detailed diagram with the components and connections used in the experimental setup is shown in Figure 6c. The process starts when the outside block card (a) generates the signal that drives the power amplifier (b), which is applied to the outside block transducer (c). This signal is then converted into ultrasound, which propagates within the acoustic channel. The acoustic signal reaches the inside block transducer (d) where it is converted into an electric signal in order to power supply the inside block card (e) and the temperature and pressure sensor (f). Data from the sensor can be read and interpreted by the inside block card (e), these data are FSK-modulated by the inside block card (e). The FSK signal is applied to the inside block transducer (d), altering the amount of acoustic reflection for the ultrasound waves that come from the outside block transducer (c). The outside block transducer (c) together with external filter (g), in turn, interpreted it as an FSK signal, which can be demodulated by the outside block card (a). The demodulated data are sent to a computer (h) so that it can be displayed.

**Figure 7:**
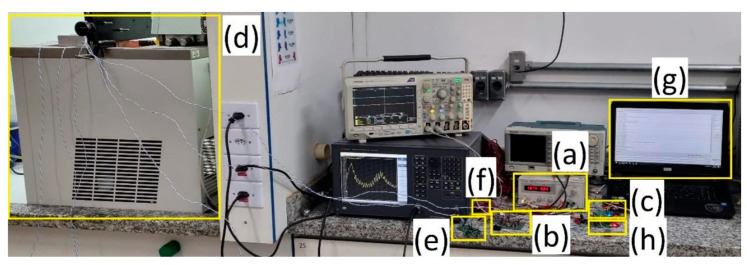
Complete system in operation for the acoustic channel formed by the combination of layers of multiple materials highlighting its main components: (**a**) bench power supply; (**b**) power amplifier; (**c**) outside block modem board; (**d**) acoustic channel in the water recipient; (**e**) inside block modem board; (**f**) pressure and temperature sensor; (**g**) computer displaying the transmitted data; (**h**) band-pass filter.

Figure 7 shows the complete system in operation, with data being received by a computer. The following parts are highlighted. Power supply (a): powers the outside block circuit and the power amplifier. Power Amplifier (b): amplifies the generated carrier with the outside block circuit, which drives the outside transducer block. Outside block circuit (c): receives data from the acoustic channel and sends it to the computer. Acoustic channel (d): formed by two 5 mm-thick steel plates and a 10 mm fluid composed of water + PARAFLU^®^ (Paraflu, São Sebastião do Caí, Brazil), in a 5:1 ratio. Inside block circuit (e): reads the pressure sensor and temperature, modulates the data in the form of an FSK signal and sends data to the acoustic channel. Pressure and temperature sensor (f). Computer (g): displays data coming from the outside block circuit. External filter circuit (h): filters the signal coming from the outside transducer block to be applied to the outside block circuit.

**Figure 8:**
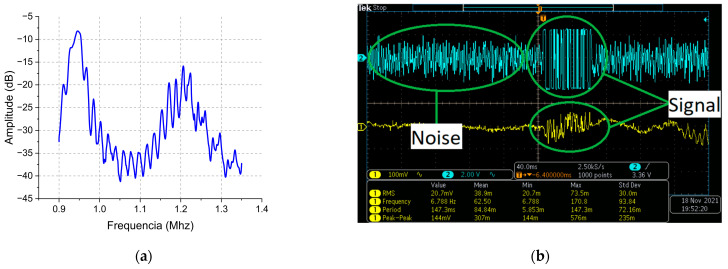
(**a**) Frequency selectivity response for the acoustic channel measured by the insertion loss with an E5063A network analyzer. (**b**) Acquired signal at the input of the electronic circuit of the outside block circuit, highlighting the noise. The yellow curve is the signal present on the outside block transducer and the blue curve is the signal at the output of the outside block comparator.

Initially, the S12 parameter of the channel was measured with a vector network analyzer (VNA), Model E5063A from HP, by connecting the transducers assembled in the system to the input and output of the VNA. This parameter measures the transmission level from one transducer to the other through the acoustic channel. Part of the energy injected in the first transducer is lost due to, among other factors, damping and differences in the acoustic impedances of the layer, which are overall measured by the insertion loss. The insertion loss of the acoustic channel is shown in Figure 8a. As can be seen, it presents a low value, with a maximum of −8.19 dB at 946.58 kHz. Due to the high insertion loss, the system was unable to operate at first. The signal at the input port of the outside block comparator is shown in Figure 8b. As can be seen, it is very noisy (highlighted by a noise label), being, therefore, a challenge to demodulate the data coming from the inside block. In order to overcome noise effects, a multiple-feedback 8th-ordered Butterworth band pass filter was used in the experiment setup. The filter parameters (45 kHz central frequency, start of rejection band at 100 kHz, maximum gain in cut-band of −40 dB and maximum band pass width of 25 kHz) were empirically fine-tuned in order to provide the adequate signal-to-noise ratio for operation. Once the signal was adequately filtered and detected, the system was stable, operating for 2 h and 30 min, with an error of 12% in data reception; the automatic gain and carrier controls were disabled during this first test.

**Figure 9:**
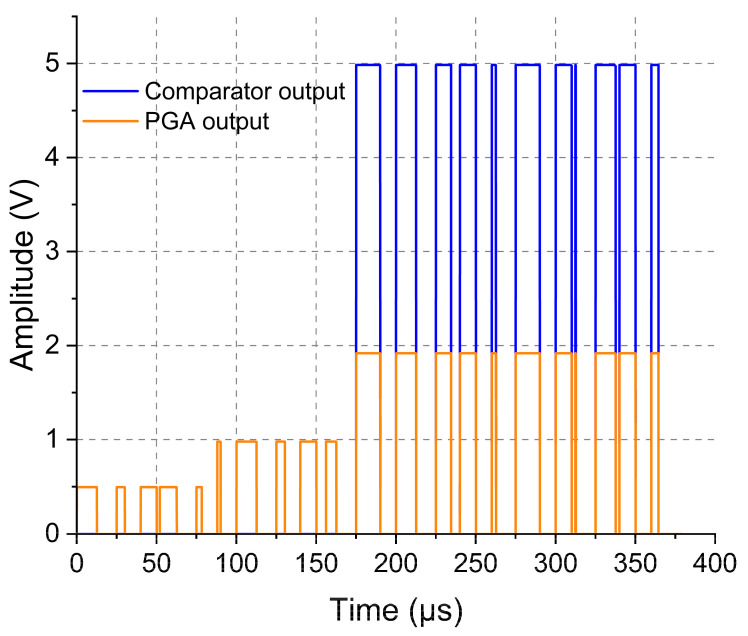
Automatic gain control actuation, obtained with a DSO1072B oscilloscope. Signal outputted from the PGA applied to the voltage comparator input with some increases in magnitude due to the gain applied by the PGA, in orange; and the signal at the output of the voltage comparator in blue. The orange level increases due to the PGA gain when the signal achieves the preconfigured comparator-specific threshold level. The blue line indicates the exact instant when this level is reached and then the signal can be decoded.

In order to assess the automatic gain control system, the circuit was initially turned on in its default configuration, with unity PGA gain. Figure 9 shows the signal at the output of the PGA, or the input of the voltage comparator, orange line, and the signal at the output of the voltage comparator of the outside block circuit, blue line. When the gain applied by the PGA was sufficient to reach the comparator voltage configured threshold, its output could saturate to indicate a high-level signal. This change can be observed in the blue line at 162.7 µs. As can be seen, the algorithm detected that the FSK-modulated signal was locked at logic zero until approximately 88.1 µs. Thus, the microcontroller (F-2) in the outside block sent an increase-gain command to the PGA, which switches from a unitary gain to a two-fold gain (at 88.1 µs), followed by a further increase to a four-fold gain (at 162.7 µs), resulting in 2 V in the output of the PGA. It is also possible to notice in Figure 9 that the comparator output signal, blue line, only follows the levels at the input when the amplitude was high enough to be detected by the comparator’s internal circuitry, which happened at 162.7 µs. Once the signal was detected, the system operated stably with the fixed gain equal to four, for 2 h and 30 min, presenting an error of 5% in the data reception. The automatic carrier control was disabled during this test.

**Figure 10:**
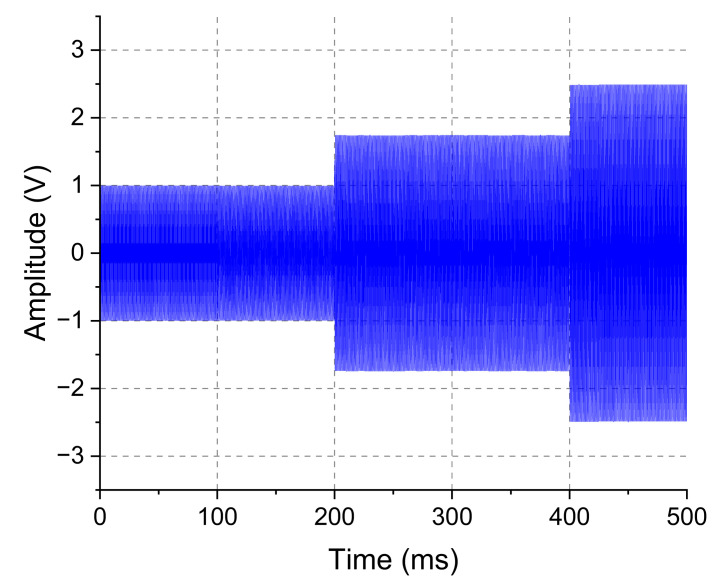
Gradual increase of carrier level. Signal sampled over the outside block transducer terminals obtained with a DSO1072B oscilloscope. It presents the increment of the output voltage on the power amplifier. The increase or decrease in the transmission carrier power is ruled by the algorithm present in Figure 3.

**Figure 11:**
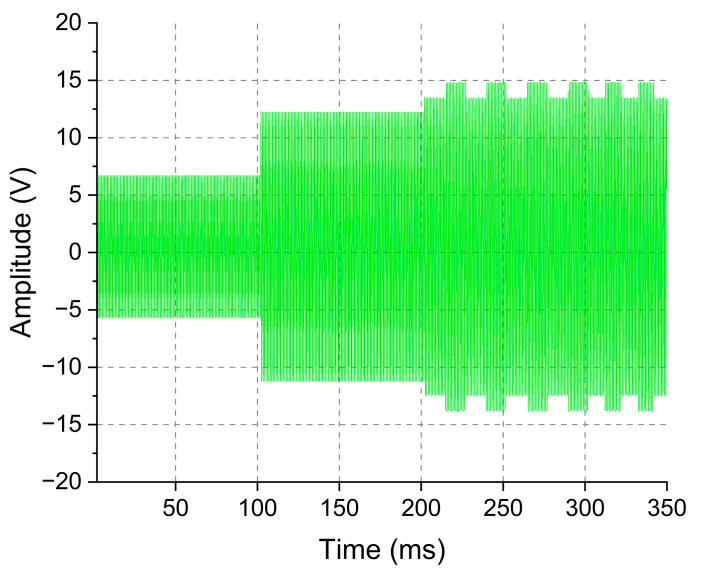
The last actuation of the automatic carrier control. Signal sampled over the outside block transducer terminals obtained with a DSO1072B. It is possible to observe that, after a few interactions, the algorithms can achieve a transmission power level sufficient to power the inside block and the transmitted data start to appear at the outside block signal.

Next, the automatic carrier control was evaluated. For this test, the system was programmed to start its operation providing the smallest possible carrier amplitude, namely, 12 V and then to increase it as required by the inside block circuit. Figure 10 shows the signal at the outside block transducer (blue line), which was time zero, the moment at which circuits were energized. As soon as the system was powered up, the algorithm gradually increased the amplitude of the carrier. After some interactions, the algorithm reached an output value of carrier amplitude that was enough to energize the inside block and it started to transmit data. Figure 11 shows the last interaction of the algorithm, increasing the amplitude of the carrier. It is possible to observe that the FSK modulation pulses appear after 200 ms, indicating that the amplitude supplied by the power amplifier circuit to the outside block transducer reached a voltage level sufficient to meet the needs of the inside block circuit and it started to work accordingly.

With the automatic carrier control algorithms, the circuit operated for 2 h and 30 m, with a 15% error rate on data reception. Automatic gain control was disabled during this test. Despite an increase from 12 to 15% in the error rate, the circuit presented a significant benefit of reducing the total energy consumption, from 2.6 W to 1.2 W when compared to the system without any control. The average consumption of the inside block was approximately 66 mW.

The proposed algorithms’ performance is summarized in Table 1, where it is possible to observe that the implementation of the proposed techniques to reduce transmission errors by controlling reception gain and the carrier level led to a reduction in the overall energy consumption of the system. The presented new approach significantly reduced both the energy consumption and the transmission error rate.

## 5. Conclusions

This paper presented an investigation on the use of frequency modulation to carry out data transmission in an acoustic channel composed of two metallic layers separated by a fluid layer. An electronic circuit capable of simultaneously transmitting data and energy between the two sides of the acoustic channel was developed. The transferred energy was able to feed a pressure and temperature sensor and a microcontroller responsible for transmitting the data through the acoustic channel at a rate of 19,200 bps. The acoustic channel consisted of two steel layers separated by a fluid layer, transmitting data and energy simultaneously. The transmission rate represents a two-fold increase in relation to previous work [1]. Two new control schemes for the acoustic transmission systems were proposed and implemented and evaluated; namely, automatic gain control and automatic control of the transmitted carrier.

These controls contributed to a significant reduction in energy consumption, meaning greater system efficiency or a lower data transmission error rate. The developed system presented a data transfer error rate of 5% when using the innovative automatic gain control, representing an improvement of more than 50% compared to the system with the control deactivated. The carrier control allowed the system to operate with an average total consumption of 1.2 W, representing a 53.15% reduction, keeping the same data transfer rate.

The results obtained herein indicate that, even under relatively high insertion loss, which is common in acoustic channels composed of multiple layers and due to inherent damping, the proposed system was able to successfully operate and thus has strong potential to be used in real applications, such as monitoring the cementing of oil wells, where the so-called inside block of the system is inaccessible through several layers of materials. Future studies will concern improving the SNR and transmitted power of wireless communication systems. One potential approach to increase the energy received by the inside block circuitry is to implement impedance-matching techniques. Additionally, SNR can be improved by a similar automatically tuned band-pass filter or by using a high-resource microcontroller such as a 32-bit one, instead of an 8-bit microcontroller as used herein, whereby, with more processing capacity, the precision of the decodification algorithm should increase and, consequently, demodulation errors should decrease.

## Figures and Tables

**Table 1:** Comparison between the tests performed with either the automatic carrier or gain control activated.

Automatic Carrier Control	Automatic Gain Control	Test Duration	Demodulation Error	Power Consumption
disabled	disabled	2 h 30 min	12%	2.6 W
disabled	enabled	2 h 30 min	5%	2.6 W
enabled	disabled	2 h 30 min	15%	1.2 W

## Data Availability

Not applicable.
